# Supplementary material for: COZOID: contact zone identifier for visual analysis of protein-protein interactions
Source: BMC Bioinformatics. 2018 Apr 6;19:125. doi: 10.1186/s12859-018-2113-6 (PMC5889581; doi:10.1186/s12859-018-2113-6)
Supplement: Supplementary file 2 — Software build. Executable binary file of the software tool. (ZIP 118,784 kb) [file 12859_2018_2113_MOESM2_ESM.zip › cozoid/java/docs/beansbinding-1.2.1-doc.zip!/org/jdesktop/el/class-use/Expression.Result.Type.html]

Uses of Class org.jdesktop.el.Expression.Result.Type (Beans Binding)


---


|  |  |  |
| --- | --- | --- |
| **Overview** \| **Package** \| **Class** \| **Use** \| **Tree** \| **Deprecated** \| **Index** \| **Help** | |  |
| PREV   NEXT | **FRAMES**    **NO FRAMES**     **All Classes** |  |


---


## **Uses of Class org.jdesktop.el.Expression.Result.Type**

| Packages that use Expression.Result.Type | |
| --- | --- |
| **org.jdesktop.el** | Customized version of EL for Beans Binding; not for general use. |

Uses of Expression.Result.Type in org.jdesktop.el

| Methods in org.jdesktop.el that return Expression.Result.Type | |
| --- | --- |
| `Expression.Result.Type` | `Expression.Result.getType()` |
| `static Expression.Result.Type` | `Expression.Result.Type.valueOf(java.lang.String name)`             Returns the enum constant of this type with the specified name. |
| `static Expression.Result.Type[]` | `Expression.Result.Type.values()`             Returns an array containing the constants of this enum type, in the order they are declared. |

Constructors in org.jdesktop.el with parameters of type Expression.Result.Type

`Expression.Result(Expression.Result.Type type,
java.lang.Object result,
java.util.List<Expression.ResolvedProperty> resolvedProperties)`

---


|  |  |  |
| --- | --- | --- |
| **Overview** \| **Package** \| **Class** \| **Use** \| **Tree** \| **Deprecated** \| **Index** \| **Help** | |  |
| PREV   NEXT | **FRAMES**    **NO FRAMES**     **All Classes** |  |


---
